# Identification of Disease-Associated Variants by Targeted Gene Panel Resequencing in Parkinson's Disease

**DOI:** 10.3389/fneur.2020.576465

**Published:** 2020-09-29

**Authors:** Kensuke Daida, Manabu Funayama, Yuanzhe Li, Hiroyo Yoshino, Arisa Hayashida, Aya Ikeda, Kotaro Ogaki, Kenya Nishioka, Nobutaka Hattori

**Affiliations:** ^1^Department of Neurology, Juntendo University School of Medicine, Tokyo, Japan; ^2^Research Institute for Diseases of Old Age, Graduate School of Medicine, Juntendo University, Tokyo, Japan; ^3^Center for Genomic and Regenerative Medicine, Graduate School of Medicine, Juntendo University, Tokyo, Japan

**Keywords:** next generation sequencing, panel resequencing, genetic association study, Parkinsion's disease, missing heritability

## Abstract

**Background:** Recent advanced technologies, such as high-throughput sequencing, have enabled the identification of a broad spectrum of variants. Using targeted-gene-panel resequencing for Parkinson's disease (PD)-associated genes, we have occasionally found several single-nucleotide variants (SNVs), which are thought to be disease-associated, in PD patients. To confirm the significance of these potentially disease-associated variants, we performed genome association analyses, using next-generation target resequencing, to evaluate the associations between the identified SNVs and PD.

**Methods:** We obtained genomic DNA from 766 patients, who were clinically diagnosed with PD, and 336 healthy controls, all of Japanese origin. All data were analyzed using Ion AmpliSeq panel sequences, with 29 PD- or dementia-associated genes in a single panel. We excluded any variants that did not comply with the Hardy–Weinberg equilibrium in the control group. Variant frequencies in the PD and control groups were compared using PLINK. The identified variants were confirmed to a frequency difference of *P* < 0.05, after applying the Benjamini–Hochberg procedure using Fisher's exact test. The pathogenicity and prevalence of each variant were estimated based on a public gene database.

**Results:** We identified three rare variants that were significantly associated with PD: rs201012663/rs150500694 in *SYNJ1* and rs372754391 in *DJ-1*, which are intronic variants, and rs7412 in *ApoE*, which is an exonic variant. The variants in *SYNJ1* and *ApoE* were frequently identified in the control group, and rs201012663/rs150500694 in *SYNJ1* may play a protective role against PD. The *DJ-1* variant was frequently identified in the PD group, with a high odds ratio of 2.2.

**Conclusion:** The detected variants may represent genetic modifiers or disease-related variants in PD. Targeted-gene-panel resequencing may represent a useful method for detecting disease-causing variants and genetic association studies in PD.

## Introduction

Parkinson's disease (PD) is the second-most frequent neurodegenerative disorder, associated with motor and non-motor symptoms (1). Clinical symptoms are characterized by tremor, rigidity, bradykinesia, and gait disturbances. To date, advanced genetic methods have revealed several genes associated with both familial and sporadic PD (2). Initially, genes were identified based on large pedigrees associated with Mendelian forms of PD, using positional cloning and linkage analyses, which resulted in the identification of *SNCA* (3), *LRRK2* (4, 5), *PRKN* (6), and *PINK1* (7). Later, next-generation sequencing (NGS) was used to identify additional causative genes, such as *ATP13A2* (8), *CHCHD2* (9), *VPS13C* (10), and *PSAP* (11). Furthermore, genome-wide association studies (GWASs) have identified single-nucleotide variants (SNVs) and other rare variants associated with sporadic PD (12–14). The explained heritability ranged from 16 to 36%, even in the latest large GWAS, as reported in 2019 (15).

Many genome-association studies, including GWAS, have been conducted for PD; however, some unrevealed genetic background remains, referred to as “missing heritability” (13, 14, 16). Missing heritability is the difference between heritability estimated from twin studies and GWAS, as GWAS has only been able to detect some of the heritability estimated from twin studies (17). Many explanations for missing heritability have been proposed, including unrevealed variants with smaller effects, rarer variants that are poorly detected by the currently available genotyping arrays, copy number variants that cannot be detected by available arrays, and the low power to detect gene–gene interactions (16). Variants of *GBA* are known to be strong risk factors for sporadic PD but have not been detected by GWAS, likely due to a low minor allele frequency.

We have developed a targeted-gene-panel resequencing protocol to screen 29 PD-associated genes, simultaneously. Panel resequencing has both advantages and disadvantages because it can identify multiple types of variants, including pathogenic variants, risk-associated variants, and rare variants of uncertain significance. Therefore, determining which variants are disease-associated can be difficult. A previous report describing Mendelian genes showed that rare functional variants occurred more frequently in sporadic PD cases than in control cases, indicating that Mendelian genes may be associated not only with familial PD but also with sporadic PD, which may be assessable using panel resequencing (18). In our analyses, through targeted-gene-panel resequencing, rare variants were identified in ~40% of PD patients with a family history or early-onset PD (data not shown), and pathogenic variants were found in an even smaller percentage of patients. We also identified several putative disease-associated variants in PD patients. We hypothesized that these variants may play a role in PD onset and could account for some degree of missing heritability. Thus, we aimed to implement target-panel resequencing, to identify associations between SNVs and familial or early-onset PD. Our method contributes to expanding the understanding of missing heritability among familial and early-onset PD patients.

## Materials and Methods

### Participants

The present study was approved by the ethics committee of Juntendo University, Tokyo, Japan, and all participants provided written informed consent to participate in the genetic research. We collected DNA samples from the Juntendo PD DNA bank, which included 766 patients with PD, who were clinically diagnosed using standard criteria (1), and 336 healthy control subjects. Among these, 407 PD patients had a family history of PD (average age at onset: 54.6 ± 15.77 years, range 6–88), and the remaining 359 PD patients were without family history (average age at onset: 42.0 ± 11.22 years, range 9–83). We also collected data regarding the Hoehn and Yahr stages for each PD patient. The healthy controls were defined as individuals without any individual or family history of neurodegenerative disorders. An overview of the clinical characteristics of the included PD patients and healthy controls is shown in Table 1.

**Table 1:** Demographic data of the analyzed subjects.

	**PD patients**	**Controls**
Total number of subjects	766	336
Gender (female:male)	366:400	114:222
Average age at onset	48.6 ± 15.35	NA
Average age at examination	57.0 ± 14.14	62.2 ± 16.36
Hoehn and Yahr stage (On phase)	2.32 ± 1.06	NA
Hoehn and Yahr stage (Off phase)	3.09 ± 1.83	NA
Subjects with known pathogenic mutations	61	0
Subjects with family history	407	NA
Average age at onset	54.6 ± 15.77	NA
Average age at examination	62.6 ± 13.57	NA
Subjects without family history	359	NA
Average age at onset	42.0 ± 11.22	NA
Average age at examination	50.7 ± 11.88	NA

*PD, Parkinson's disease; NA, not applicable*.

*The data are presented as the mean ± standard deviation*.

### Processing Data Output From the Ion Torrent System

The sequencing analysis of the Ion AmpliSeq panel (Thermo Fisher Scientific, Waltham, MA, USA) was performed using the Ion Chef System (Thermo Fisher Scientific) and the Ion S5 Sequencer (Thermo Fisher Scientific), according to the manufacturer's instructions. Our Ion AmpliSeq panel (Thermo Fisher Scientific, IAD103177_182) included 29 PD- and dementia-related genes (Table 2), and its coverage was 98.34% (829 amplicons, missed: 1,646 bp) (manuscript in preparation). The output data were obtained as a variant call format (VCF) file from the Ion torrent system. VCF files were processed using vcftools (19).

**Table 2:** PD- and dementia-related genes analyzed by resequencing.

**Genes related to PD**	**Genes related to dementia**
*SNCA (PARK1,4)*	*MAPT*
*parkin (PARK2)*	*PSEN1*
*UCH-L1 (PARK5)*	*GRN*
*PINK1 (PARK6)*	*APP*
*DJ-1 (PARK7)*	*APOE*
*LRRK2 (PARK8)*	
*ATP13A2 (PARK9)*	
*GIGYF2 (PARK11)*	
*HTRA2 (PARK13)*	
*PLA2G6 (PARK14)*	
*FBXO7 (PARK15)*	
*VPS35 (PARK17)*	
*EIF4G1 (PARK18)*	
*DNAJC6 (PARK19)*	
*SYNJ1 (PARK20)*	
*DNAJC13 (PARK21)*	
*CHCHD2 (PARK22)*	
*VPS13C (PARK23)*	
*GCH1*	
*NR4A2*	
*RAB7L1*	
*BST1*	
*C19orf12*	
*RAB39B*	

### Statistical Analysis to Compare the Frequencies of Non-rare Variants

**Figure 1:**
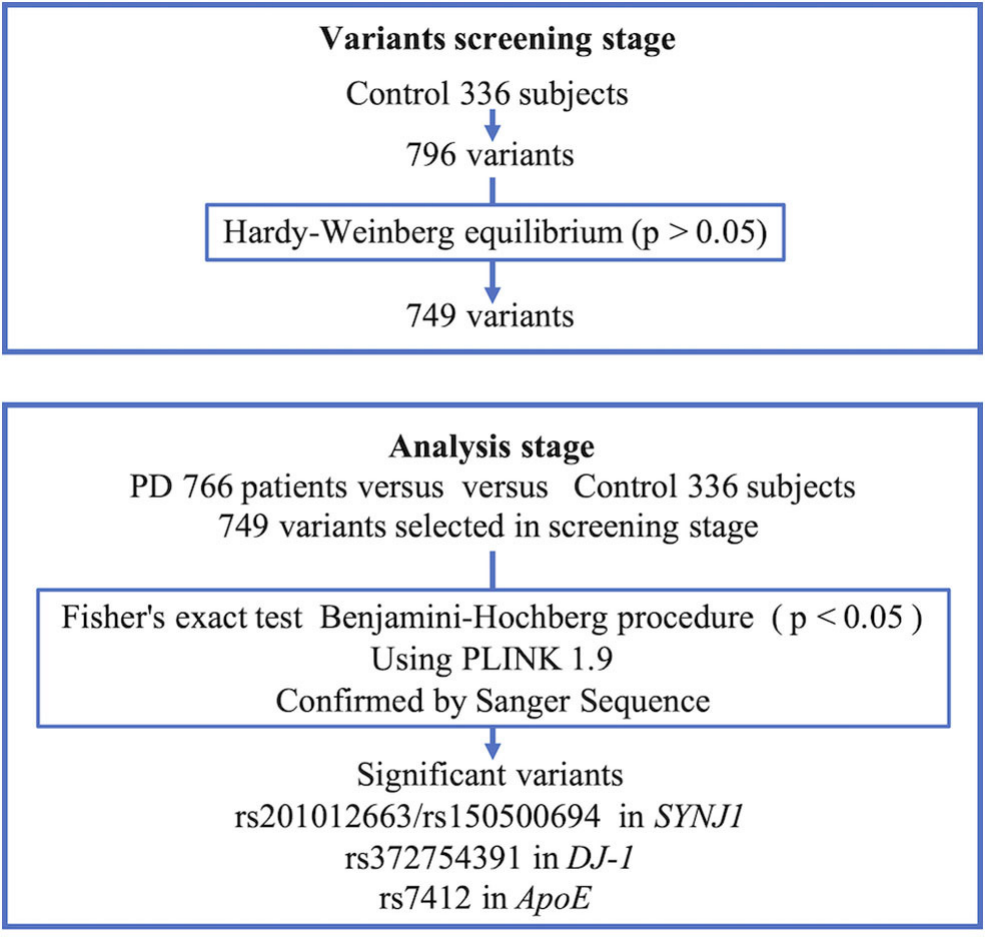
Scheme of analysis. PD, Parkinson's disease; *SYNJ1*, Synaptojanin 1; *APOE*, Apolipoprotein E.

We confirmed all samples with a mean depth > 100 and excluded those amplicons with read depths smaller than 10. The analyzed variants were confirmed to exist among the target sequences and to have read depth of coverages >45. We also calculated the coverage percentage. During the variant-screening stage, we excluded all variants that did not comply with Hardy–Weinberg equilibrium (HWE; *P* < 0.05) within the control group (Figure 1). We analyzed only the control group during the variant-screening stage because performing HWE analysis while including PD patients would introduce bias. During the analysis stage, the variant frequencies observed for the PD and healthy non-PD groups were compared using PLINK 1.9 (20). To verify this comparison, variants with a frequency difference of *P* < 0.05, based on the performance of the Benjamini–Hochberg procedure and Fisher's exact test, were analyzed using the genotyping data available in 4.7KJPN, from the Japanese Multi Omics Reference Panel (jMorp) (21), and a genome aggregation database (gnomAD) (22). The scheme used for the analysis is presented in Figure 1. To confirm the presence of significant variants identified during the association study, we conducted Sanger sequencing on three cases with the variant and three cases without the variant, during the panel resequencing experiment.

## Results

The percentage of coverage was calculated, showing that 99.7% of the total dataset was read at a depth of least 1×, 98.9% at 20×, 97.9% at 100×, and 84% at 500×. During the variant screening stage, we identified 796 variants in our healthy controls, of which 749 were retained after screening for HWE compliance (*P* < 0.05) and were included in the association analysis performed using PLINK. We conducted Sanger sequencing on nine significant variants with *p*-values below 0.05 after performing the Benjamini–Hochberg procedure for Fisher's exact test, and five of them (chr1:65830299 T>G, chr1:65830300 T>G, chr3:184033555, chr2:233620927-233620929, and chr1:205743943) were not validated and excluded from the analysis. All of the false-positive variants were positioned around the tandem repeat of mononucleotides that was considered to cause false positives. Table 3 shows the top 15 variants that had the lowest *p-*values based on Fisher's exact test.

**Table 3:** Top 15 variants detected in genome association studies.

**Ranking**	**Position**	**rs number**	**Gene**	**AF in PD**	**AF in control**	**UNADJ**	**GC**	**BONF**	**SIDAK_SS**	**SIDAK_SD**	**FDR_BH**	**Confirmed by Sanger sequencing**
1	chr21:34050937	rs201012663	*SYNJ1*	0.2004	0.4048	1.06E-23	1.66E-08	7.76E-21	INF	INF	7.76E-21	Yes
1	chr21:34050941	rs150500694	*SYNJ1*	0.2004	0.4048	1.06E-23	1.66E-08	7.76E-21	INF	INF	7.76E-21	Yes
3	chr1:8029508-8029510	rs372754391	*DJ-1*	0.09465	0.04464	6.41E-05	0.02459	0.04708	0.04599	0.04563	0.006726	Yes
4	chr19:45412079	rs7412	*APOE*	0.01762	0.04315	0.0004541	0.04863	0.3337	0.2838	0.2815	0.04172	Yes
5	chr1:17328732	NA	*ATP13A2*	0	0.00744	0.0007247	0.05734	0.5327	0.4131	0.4097	0.05919	N/A
6	chr22:32887150	rs9726	*FBXO7*	0.2454	0.186	0.002182	0.08487	1	0.7993	0.7953	0.1316	N/A
7	chr21:34037203	rs142813430	*SYNJ1*	0.003264	0.01488	0.002259	0.08592	1	0.8103	0.8059	0.1316	N/A
8	chr3:132221076	rs11293788	*DNAJC13*	0.03264	0.01042	0.002481	0.08886	1	0.8389	0.8345	0.1316	N/A
9	chr3:132194001	rs11920646	*DNAJC13*	0.06462	0.03274	0.002485	0.08892	1	0.8394	0.8345	0.1316	N/A
10	chr1:17323061	NA	*ATP13A2*	0	0.005952	0.002507	0.08919	1	0.8419	0.8367	0.1316	N/A
11	chr1:65830301	rs372128441	*DNAJC6*	0.01828	0.002976	0.004319	0.1085	1	0.9585	0.9559	0.21	N/A
12	chr21:27369838	rs2051503	*APP*	0.1012	0.1429	0.00457	0.1108	1	0.9655	0.9631	0.21	N/A
13	chr12:40758652	rs371863	*LRRK2*	0.4204	0.4836	0.005873	0.1214	1	0.9868	0.9855	0.2539	N/A
14	chr21:27348447	rs3737414	*APP*	0.3551	0.4152	0.007258	0.1311	1	0.9953	0.9946	0.2835	N/A
15	chr15:62226277	rs1009641857	*VPS13C*	0.06919	0.04018	0.008459	0.1387	1	0.9981	0.9977	0.2835	N/A

*UNADJ, Unadjusted, asymptotic significance value; GC, Genomic control-adjusted significance value; BONF, Bonferroni-adjusted significance value; SIDAK_SS, Sidak single-step-adjusted significance value; SIDAK_SD, Sidak step-down-adjusted significance value; FDR_BH, Benjamini and Hochberg (1995) step-up FDR control; N/A, not applicable*.

Four variants were significantly associated with PD: rs201012663 and rs150500694 in *SYNJ1*, rs372754391 in *DJ-1*, and rs7412 in *ApoE* (Table 4). The two *SYNJ1* variants, rs201012663 and rs150500694, were considered to represent a single variant because they are located four bases apart and demonstrated the same frequency in our subjects and public gene databases, which suggests that these variants are strongly linked (Tables 3, 4). The *SYNJ1* variants are both located in an intron, with an odds ratio of 0.37. The *DJ-1* variant (rs372754391) was also intronic and was more frequently identified in the PD cohort than in controls, with an odds ratio of 2.2. However, its frequency in the public database was quite large compared with the frequency in our data. The *ApoE* variant was exonic and was more frequently observed in the control group than in the PD group, with an odds ratio of 0.39. The *ApoE* variant was one of the single-nucleotide polymorphisms (SNPs) that determine the *ApoE* genotype. The E2 *ApoE* genotype was more frequently observed in the control group, whereas the E4 genotype was more frequently observed in the PD group (Table 5). No significant differences in age, age at onset, or Hoehn and Yahr scores were observed between patients with and without detected variants (Table 6).

**Table 4:** Details of the detected variants with significant value.

**Gene symbol**	***SYNJ1***	***DJ-1***	***APOE***
dbSNP153	rs201012663 rs150500694	rs372754391	rs7412
Position	chr21:34050937-34050941	chr1:8029508-8029510	chr19:45412079
Reference/alternative	AATATA/AATT	GGG/GAA	C/T
Exon or intron	Intron	Intron	Exon
Allele frequency in PD	0.2004 (307/1,532)	0.09465 (145/1,532)	0.01762 (27/1,532)
Genotype in PD (alt/alt, alt/ref, ref/ref)	70, 167, 529	4, 137, 625	0, 27, 739
Allele frequency in control	0.4048 (272/672)	0.04464 (30/672)	0.04315 (29/672)
Genotype in control (alt/alt, alt/ref, ref/ref)	61, 150, 125	1, 28, 307	0, 29, 307
Odds ratio	0.367	2.237	0.398
gnomAD 2.1 EAS	NA	NA	0.07511 (947/12,608)
gnomAD 3.0 EAS	0.4181 (1,297/3,102) 0.4178 (1,297/3,104)	0.6263 (1904/3040) 0.6352 (1,936/3,048)	0.08029 (251/3,126)
jMorp	0.4257 0.4257	NA	0.044
*p*-value (PD vs. gnomAD 3.0)	<0.001	<0.001	<0.001
*p-* value (control vs. gnomAD 3.0)	0.2767	<0.001	0.0003

*SYNJ1, Synaptojanin 1; APOE, Apolipoprotein E; PD, Parkinson's disease; gnomAD, genome aggregation database; EAS, east Asia; NA, not applicable; jMorp, Japanese Multi Omics Reference Panel; dbSNP, the Single Nucleotide Polymorphism Database; alt, alternative allele; wild, reference allele*.

*The variant in SYNJ1 is recorded separately as rs201012663 and rs150500694 in dbSNP153, in gnomAD, and in jMorp*.

*The variant in DJ-1 is recorded as rs372754391 in dbSNP153 and registered separately as two single variants in gnomAD*.

**Table 5:** Allele frequencies in patients, according to *APOE* genotype.

	**PD (*n* = 1,532)**	**Controls (*n* = 672)**	
**Genotype of *APOE***	**Allele frequency (%)**	**Allele** **frequency (%)**	***p*-value (PD vs. control)**
E1	0	0	NA
E2	1.76	4.32	0.0006
E3	86.88	87.5	0.3728
E4	11.36	8.18	0.0137

*E, epsilon; PD, Parkinson's disease; NA, not applicable; APOE, Apolipoprotein E*.

**Table 6:** Clinical characteristics of patients, according to the presence of the identified variants.

	**Presence of the variant**	**Age**	**Age at onset**	**Hoehn and Yahr scale**	**Disease duration (years)**
*SYNJ1 rs201012663 rs150500694*	+	58.0 ± 17.73	48.5 ± 15.67	2.3 ± 1.11	9.35 ± 9.18
	–	56.6 ± 15.28	48.7 ± 15.22	2.33 ± 1.05	8.01 ± 8.14
	*p*-value	0.209200773	0.96096438	0.81537244	0.05363004
*DJ-1 rs372754391*	+	57.0 ± 15.06	49.9 ± 15.48	2.41 ± 1.11	7.07 ± 6.96
	–	57.0 ± 24.72	48.4 ± 23.42	2.30 ± 1.06	8.73 ± 8.78
	*p*-value	0.972443834	0.304154621	0.315455549	0.016077725
*APOE rs7412*	+	58.8 ± 14.44	49.3 ± 15.92	2.10 ± 0.88	9.48 ± 9.75
	–	57.0 ± 14.14	48.7 ± 15.25	2.33 ± 1.07	8.39 ± 8.45
	*p*-value	0.60125477	0.84599687	0.22211054	0.57046758

*SYNJ1, Synaptojanin 1; APOE, Apolipoprotein E*.

*Data are presented as the mean ± standard deviation*.

*The p-values were calculated by Student's t-test, comparing PD patients with the variant with those without the variant*.

We do not have data for four variants (rs16856139, rs11931532, rs11931074, and rs1994090) that were previously identified in a GWAS performed in Japanese PD patients because these variants were absent from our target panel (13). *LRRK2* G2385R (rs34778348), which is a risk factor for PD in East Asian individuals, was the 21st most significant variant identified among our cohort (23). Except for rs34778348, none of the currently known risk variants for PD were detected.

## Discussion

We performed a genetic case–control analysis, using NGS data from our Ion AmpliSeq panel. We identified three variants in three different genes: the combination of rs201012663 and rs150500694 in *SYNJ1*, rs372754391 in *DJ-1*, and rs7412 in *ApoE*. None of these three variants were reported as PD-related variants when we searched a GWAS catalog on June 8, 2020 (24). Our identified variants might account for missing heritability in PD. Targeted resequencing could perform deeper reads of selected genes associated with phenotypes than the microarrays that are normally used in GWAS. Thus, targeted resequencing-based association studies may be able to identify risk variants that have not been previously identified by GWAS (17).

The three identified variants have never previously been reported as variants associated with PD. In our study, variants in *SYNJ1* (rs201012663 and rs150500694) showed a higher frequency in the control group than in the PD group. *SYNJ1* is known to be a causative gene for early-onset Parkinsonism, with atypical characteristics, such as seizures, dystonia, and dementia, with an autosomal-recessive inheritance pattern (25, 26). This gene encodes the protein Synaptojanin 1, a polyphosphoinositide phosphatase that is concentrated at synapses (27, 28). Synaptojanin 1 is associated with synaptic vesicle endocytosis. The variants identified in *SYNJ1* (rs201012663/rs150500694) in this study have not previously been reported to be pathogenic variants. Synaptojanin 1 is also known to play a role in the pathogenesis of Alzheimer's disease (AD), associated with a PI (4, 5)P_2_ imbalance. The haploinsufficiency of *SYNJ1* protects cells from the neurotoxic actions of Aβ42 (29). The variants rs201012663/rs150500694 might play a similarly protective role against alpha synuclein-mediated neurotoxicity.

The identified variant in *DJ-1* might be interesting, due to the high odds ratio of 2.2. However, this variant may be specific to ethnicity because the frequency of this variant among our healthy controls was lower than that observed in public databases. This variant was not recorded in jMorp, one of the largest genomic databases in Japan, suggesting its rarity in the Japanese population. *DJ-1* was initially identified as an oncogene and was later found to cause familial PD (30). *DJ-1* has also been associated with other disorders, including stroke, familial amyloidotic polyneuropathy, and type 2 diabetes (30–33). DJ-1 has several functions, including transcriptional regulation, antioxidative stress reactions, chaperone, protease, and mitochondrial regulation (30). DJ-1 is expressed in almost all cells, including neurons and glial cells. DJ-1 protein contains three cysteine residues, C46, C56, and C106. C106 is likely to be influenced by oxidative stress and oxidized into SOH, SO_2_H, and SO_3_H (34–36). DJ-1 containing a C106 residue that has been oxidized to SO_3_H is thought to represent an inactive form (37). In the brains of PD patients, excessively oxidized forms of DJ-1 have been observed (38). The identified mutation might facilitate oxidation, inactivating DJ-1.

*APOE* genotypes have previously been associated with an increased risk of AD (39, 40). rs7412 is one of two SNVs that have been defined in common allelic *APOE* variants. *APOE4* is known to represent a strong risk factor for AD. The variant (rs7412) identified in our study is included in *APOE1 or APOE2*, which are known to decrease the risk of AD. rs7412 was significantly rare in the PD group in our study. In our study, *APOE2* was significantly rare in the PD group, whereas *APOE4* was significantly frequent in the PD group. Larger research studies have concluded that *APOE* epsilon had no association with PD onset (41). Differences between our study and past studies may be due to the smaller sample size included in our study and differences in the ethnicities of the participants.

In our study, SNVs detected in previous GWAS were not identified in our cohort because most of the reported risk-associated SNVs have been identified in non-coding regions, which were not included in our targeted panel (42). Targeted resequencing can cover more SNVs within the targeted exons than DNA microarrays, which are commonly used in GWAS. Our method might enable the detection of SNVs in exons or near exons that are not included in the SNP chips used for GWAS. Our target panel was designed to include all exons and the 25 bp up- and downstream of the exon–intron boundaries. Therefore, our method allowed the discovery of PD-related variants that were not detected by GWAS. The inclusion of patients with a family history or early-onset PD in our cohort might facilitate the detection of susceptibility-associated variants, with deep genetic backgrounds. For example, mutations in *GBA* are more frequently identified in familial PD patients than in sporadic PD patients (43). However, our panel resequencing approach also has several disadvantages. This approach cannot be used to identify novel genes associated with PD and does not cover the majority of introns and transcriptional regulatory regions. The variants detected in this study may also be associated with sporadic PD, similar to GWASs that identified causative genes associated with sporadic PD that were previously reported to be causative genes for familial PD (*SNCA, MAPT*, and *LRRK2*) (44).

Our study includes the following limitations: (i) the sample size is too small to satisfy genome-wide significance, (ii) the lack of a second cohort to confirm our results, (iii) the possibility of sampling bias in the control group because the allele frequencies of variants in the public database were different from those identified in our healthy control group, (iv) the absence of any functional analysis to support our results, and (v) the lack of copy number variant evaluations.

We developed a new approach for surveying susceptibility-associated variants by using targeted resequencing, which may represent an effective method for revealing hidden disease-associated variants. Further studies that include additional patients remain necessary to confirm the suitability of this approach for the identification of disease-associated variants.

## Data Availability Statement

The DNA sequence data of 1,102 participants used in this study are based on the informed consent of genetic testing from all participants according to the formal procedure approved by the Juntendo University School of Medicine Ethics Committee. However, some participants have refused to publish their DNA sequence data in public databases. Therefore, if the reader wishes to use the raw data used in this paper, please request directly to the corresponding author Manabu Funayama, funayama@juntendo.ac.jp.

## Ethics Statement

The studies involving human participants were reviewed and approved by Juntendo University School of Medicine Institutional Review Board (No. 2019227). The patients/participants provided their written informed consent to participate in this study.

## Author Contributions

KD designed and performed the experiments, analyzed the data, wrote the first manuscript, and revised the manuscript. MF designed the study, wrote the first manuscript, and revised the manuscript. YL, HY, AH, AI, KO, and KN performed the experiments, analyzed the data, and revised the manuscript. NH directed the research project and revised the manuscript. All authors contributed to the article and approved the submitted version.

## Conflict of Interest

The authors declare that the research was conducted in the absence of any commercial or financial relationships that could be construed as a potential conflict of interest.
